# Wound Healing Activity of the Essential Oil of *Bursera morelensis*, in Mice

**DOI:** 10.3390/molecules25081795

**Published:** 2020-04-14

**Authors:** Judith Salas-Oropeza, Manuel Jimenez-Estrada, Armando Perez-Torres, Andres Eliu Castell-Rodriguez, Rodolfo Becerril-Millan, Marco Aurelio Rodriguez-Monroy, Maria Margarita Canales-Martinez

**Affiliations:** 1 Laboratorio de Farmacognosia, UBIPRO, Facultad de Estudios Superiores Iztacala, UNAM, Av. de los Barrios No. 1, Los Reyes Iztacala, Tlalnepantla, Edo, Mex 54090, Mexico; judithsalazo@hotmail.com (J.S.-O.); rbecerrilm85@gmail.com (R.B.-M.); 2 Instituto de Química, UNAM, Circuito Exterior, Ciudad Universitaria, Coyoacan CDMX 04510, Mexico; manuelj@unam.mx; 3 Departamento de Biología Celular y Tisular, Facultad de Medicina, Universidad Nacional Autónoma de México, Av. Universidad 3000, CDMX 04510, Mexico; armandop@unam.mx (A.P.-T.); castell@unam.mx (A.E.C.-R.); 4 Carrera de Medicina, Facultad de Estudios Superiores-Iztacala, UNAM, Av. de los Barrios No. 1, Los Reyes Iztacala Tlalnepantla, Edo, Mex 54090, Mexico; dr.marcorodriguezmonroy@gmail.com

**Keywords:** Burseraceae, essential oil, terpenes, wound healing

## Abstract

*Bursera morelensis* is used in Mexican folk medicine to treat wounds on the skin. It is an endemic tree known as “aceitillo”, and the antibacterial and antifungal activity of its essential oil has been verified; it also acts as an anti-inflammatory. All of these reported biological activities make the essential oil of *B. morelensis* a candidate to accelerate the wound-healing process. The objective was to determine the wound-healing properties of *B. morelensis’* essential oil on a murine model. The essential oil was obtained by hydro-distillation, and the chemical analysis was performed by gas chromatography-mass spectrometry (GC-MS). In the murine model, wound-healing efficacy (WHE) and wound contraction (WC) were evaluated. Cytotoxic activity was evaluated in vitro using peritoneal macrophages from BALB/c mice. The results showed that 18 terpenoid-type compounds were identified in the essential oil. The essential oil had remarkable WHE regardless of the dose and accelerated WC and was not cytotoxic. In vitro tests with fibroblasts showed that cell viability was dose-dependent; by adding 1 mg/mL of essential oil (EO) to the culture medium, cell viability decreased below 80%, while, at doses of 0.1 and 0.01 mg/mL, it remained around 90%; thus, EO did not intervene in fibroblast proliferation, but it did influence fibroblast migration when wound-like was done in monolayer cultures. The results of this study demonstrated that the essential oil was a pro-wound-healing agent because it had good healing effectiveness with scars with good tensile strength and accelerated repair. The probable mechanism of action of the EO of *B. morelensis*, during the healing process, is the promotion of the migration of fibroblasts to the site of the wound, making them active in the production of collagen and promoting the remodeling of this collagen.

## 1. Introduction

In Mexico, medicinal plants are the most valuable material resource of traditional indigenous medicine [1]. This is due to the great diversity, derived from a complex biogeographic [2], and cultural history. The *Bursera* genus, which comprises approximately 100 species, is used in Mexican folk medicine. In Mexico, it is possible to find 80 endemic species (out of a total of 84) distributed mainly in the tropical dry forest of the country [3]. These plants are characterized by an exudative resin channel system [4]. The biological effects of these species, including their cytotoxic, antiproliferative, antimicrobial, insecticidal, and anti-inflammatory activity, have been attributed to their essential oils, diterpenes, triterpenes, sterols, and lignans [4,5,6,7,8].

*Bursera morelensis* is an endemic tree of Mexico, known as “Aceitillo”, that has been reported in the treatment of skin wounds. The people of San Rafael, Coxcatlan (Puebla, Mexico) make a tea with the bark of this species to wash the wound. It has been verified that the essential oil (EO) of this species has antibacterial and antifungal activity [9,10] and also acts as an anti-inflammatory [8].

Wound healing is divided into three sequential phases, and each phase has its own time period, as well as particular tissues and cell lines [11,12,13]. The first phase is the inflammatory phase, in which a clot forms to stop the hemorrhage; then comes the vasodilation and the activation of the immune defense mechanisms [14,15]. The proliferative phase of epidermal, endothelial, and fibroblast cells is next [16], which generates initial granulation tissue [17], and angiogenesis occurs [12]. In the last phase, the granular tissue is remodeled through the generation of new collagen fibers, and differentiation of fibroblasts occurs in myofibroblasts, which increase the tensile strength and allow the approximation of the edges of the lesion [12,17].

Plants have immense potential for the management and treatment of wounds. A large number of plants are used by tribal and folklore in many countries for the treatment of wounds and burns [18]. The molecular and physiological effects of extracts and components of medicinal plants are often characterized in research studies of mammalian systems; as of 2008, 68% of all pharmaceutical products were derived from plants or inspired by plants [19,20].

The characteristics of the EO have made them highly valued in the industry for use in food, cosmetic, and pharmaceutical applications; these secondary metabolites have been related as potent antioxidants, anti-free radicals, and metal chelators, which also have anti-nociceptive, neuroprotective, anticonvulsant, and anti-inflammatory properties, reported in preclinical studies, which are characterized as possible sources for the development of new drugs [21,22,23,24].

Taking into account these biological properties, the EO of *B. morelensis* could be a potential candidate to make the wound healing process more efficient. In the present study, the wound-healing capability of the EO of this essential oil in mice was evaluated.

## 2. Results

### 2.1. Chemical Characterization of Essential Oil

The oil yield was 0.19%. The GC-MS analysis identified 18 compounds in the EO of *B. morelensis*. The main compounds were *p*-menthane (38.41%) and β-phellandrene (35.25%). Other important components were: α-pinene (8.37%), caryophyllene (5.19%), caryophyllene oxide (0.26), β-myrcene (3.6%), sabinene (3.54%), and p-cymene (2.1%) (Table 1).

### 2.2. Skin Irritation Study and Cytotoxicity

**Figure 1:**
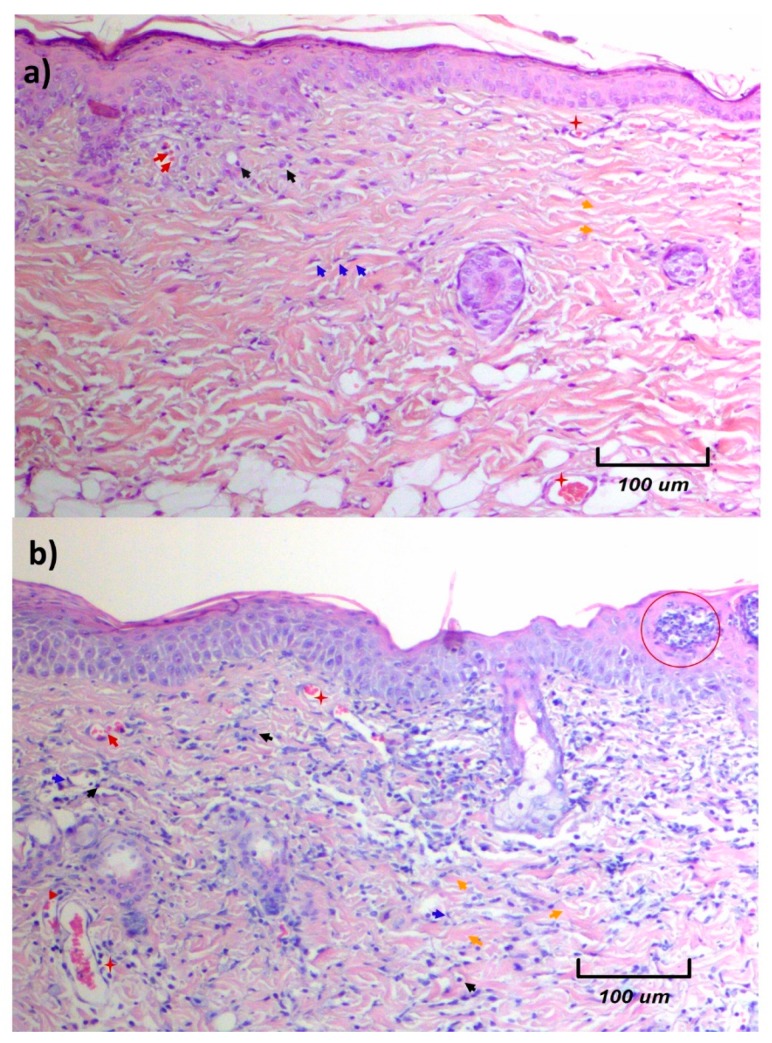
Histology of skin samples obtained from sites with different treatments on day 3. Samples were stained with hematoxylin and eosin (H&E). (**a**) Control samples of untreated healthy skin. (**b**) In healthy skin, treated with 25% *B. morelensis’* essential oil (EO), cellular detritus was observed (red circles). All photos were taken at 10× magnification. ►Erythrocytes, ►collagen fibers, ►basophil cells (positive hematoxylin), ►fibroblast, **+** blood vessel.

The 25% essential oil (25EO)-treated wounds showed slight skin redness that was detected 12 h after the first application, and the redness decreased after around 24 h. At 72 h, no skin redness was detected, but a slight peeling of the skin could be observed. Histopathological analysis of the skin of the 25EO-treated wounds showed an increased number of dead cells according to the presence of pyknotic nuclei. In addition, cellular detritus was observed on the epidermis of the skin treated with 25EO. This increased presence of cells in the treated area was probably part of a primary cellular response to this oil being recognized as a foreign agent; likewise, the presence of cellular detritus could indicate the activity of macrophages (Figure 1).

**Figure 2:**
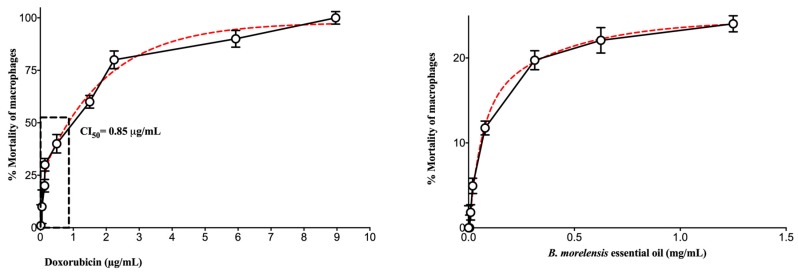
Cytotoxicity of *B. morelensis* oil compared with cytotoxicity of doxorubicin.

Regarding the cytotoxicity test, peritoneal macrophages from BALB/c mice were used. The results showed that the EO of *B. morelensis* had a mortality percentage of 24% at a concentration of 1.2 mg/mL, which was significantly lower than the half-maximal inhibitory concentration (IC_50_) of doxorubicin, which was 0.85 µg/mL. This means that the cytotoxicity of the EO was almost 1000 times less than that of doxorubicin (Figure 2).

### 2.3. Wound-Healing Efficacy (% WHE)

**Figure 3:**
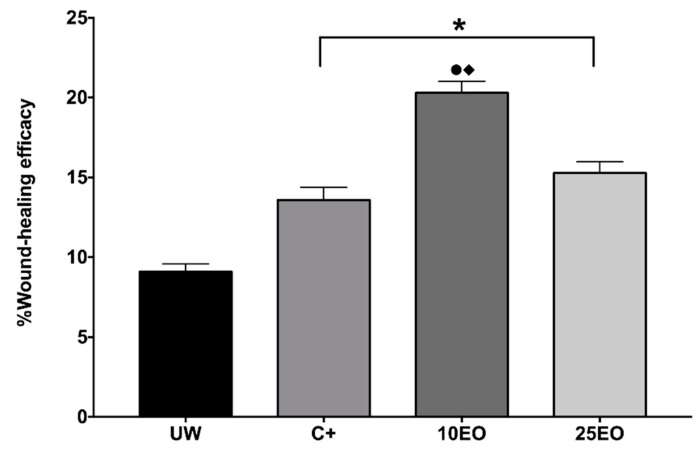
Wound-healing efficacy (% WHE). UW, untreated wound. C^+^, positive control—Recoveron NC. 10EO, 10% essential oil. 25EO, 25% essential oil. *Significant differences with respect to UW. ●Significant differences with respect to C^+^. ♦Significant differences with respect to 25EO (*p* < 0.01).

Wound resistance to the tension was measured according to the tensiometric method. We observed that untreated or healthy skin (HS) showed 28% WHE, the positive control (C^+^) showed 38%, the 10EO treatment showed 36%, and the 25EO showed 34% (Figure 3). It could be seen that the 10% essential oil (10EO) was the one with the highest WHE.

**Figure 4:**
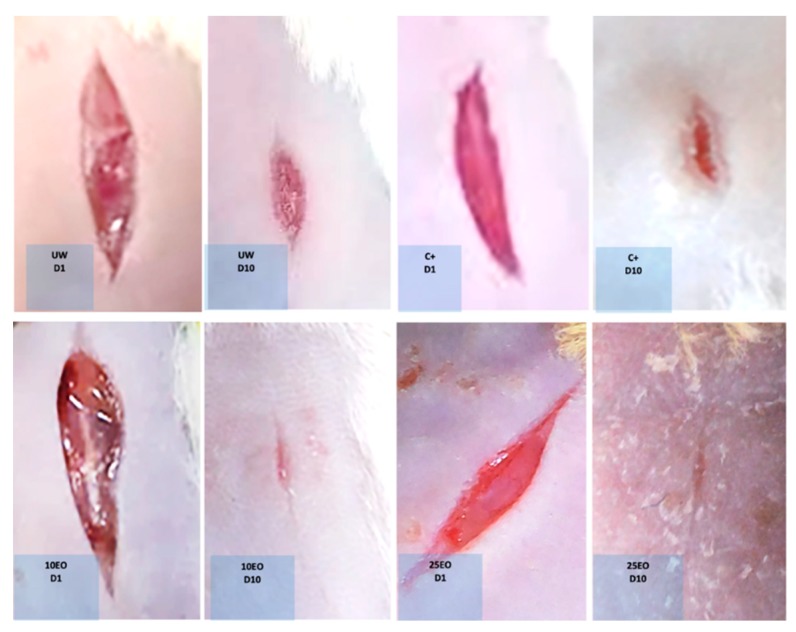
Macroscopic examination of wounds on the first and tenth day of the experiment. UW, untreated wound. C^+^, positive control—Recoveron NC. 10EO, 10% essential oil. 25EO, 25% essential oil.

Likewise, it could be observed that in mice treated with EO, in general, wounds were completely closed, while, in the other treatments, wounds did not completely close (Figure 4).

### 2.4. Incision Wound Model

**Figure 5:**
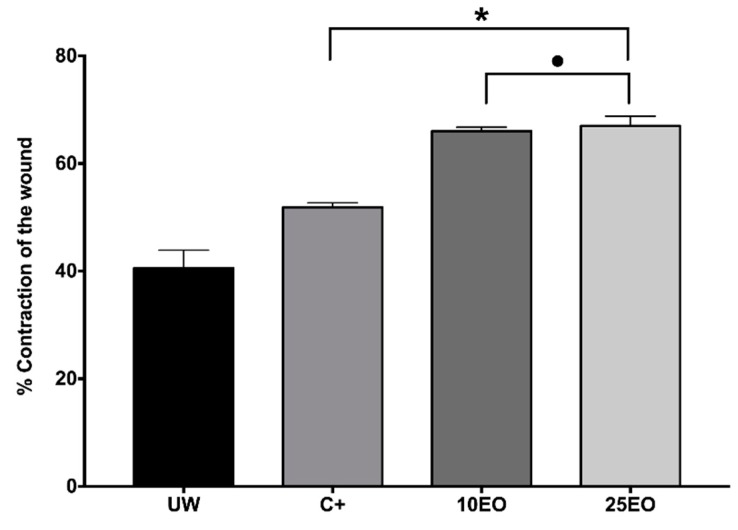
Percentage of wound contraction 10 days post-wound. UW, untreated wound. C^+^, positive control—Recoveron NC. 10EO, 10% essential oil. 25EO, 25% essential oil. * Significant differences between C^+^, 10EO, and 25EO with respect to UW. ●Significant differences with respect to C^+^ (*p* < 0.01).

In the incision wound model, it was observed that 67.2% of wounds treated with 10EO closed, and 65.3% of wounds treated with 25EO closed, while 45.01% of wounds closed in the skin with no treatment (Figure 5). 

**Figure 6:**
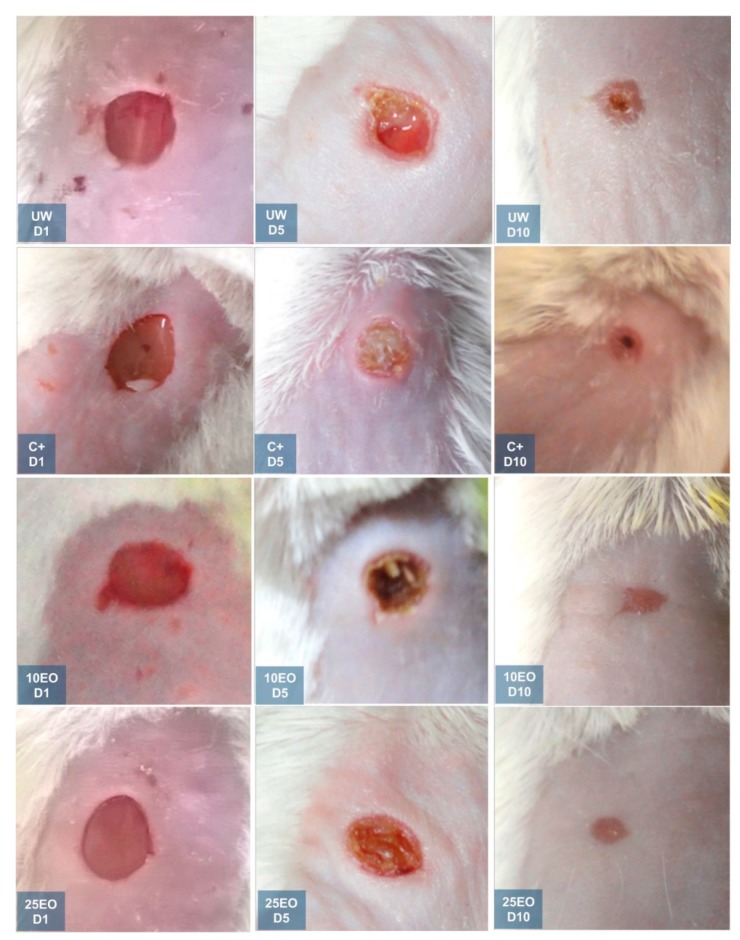
Macroscopic examination of wounds at the first, fifth, and tenth day of the experiment. UW, untreated wound. C^+^, positive control—Recoveron NC. 10EO, 10% essential oil. 25EO, 25% essential oil.

Likewise, a more homogeneous appearance of the skin was observed with both EO treatments. On day 10, in untreated mice, scabs could be observed, and, in the C^+^, mice were observed to have larger scabs (Figure 6). This coincided with the histology 10 days after treatment; on healthy skin with both stains, the three layers of skin were clearly defined, and we could distinguish hair follicles, glands, blood vessels, fibroblasts, and dark blue-purple cell nuclei; with Masson’s stain, collagen fibers were uniformly blue since they formed a uniform network/matrix where some of the fibroblasts (uniform) producing the collagen could be observed.

**Figure 7:**
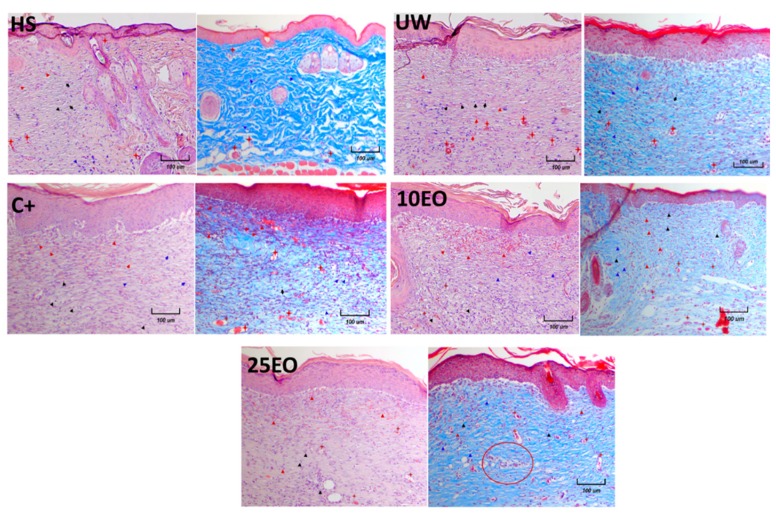
Histology of skin samples from a treated wound on day 10 of treatment. Samples were stained with hematoxylin and eosin (H&E) and Masson’s trichrome. All photos were taken at a 10X magnification. HS, healthy skin. UW, untreated wound. C^+^, positive control—Recoveron NC. 10EO, 10% essential oil. 25EO, 25% essential oil. ►Erythrocytes, ►basophil cells (positive hematoxylin), ►fibroblast, **+** blood vessel.

In the UW group, it was observed that the skin layers were restored, although they did not show accessories, such as hair follicles or glands. It was also possible to observe blood vessels, as the arrangement of collagen was lax compared to the complex arrangement that existed in healthy skin. In addition, a large number of nuclei of both fibroblasts and other cells was observed. In the C^+^ group, the epidermis and dermis were clearly differentiated, and the only skin accessories found were blood vessels. Likewise, many cell nuclei and erythrocytes were observed, and the erythrocytes were more evident with Masson’s stain, where some whitish areas were also observed, which indicated fewer collagen fibers. In both EO treatments, a better structure of scar tissue and a greater deposit of collagen were observed; likewise, different from the C+ group, fewer cell nuclei were observed, which in some points showed an arrangement similar to that of incipient glands. On the other hand, the plot was formed by the collagen fibers, a major sample, similar to that of HS (Figure 7).

### 2.5. In Vitro Tests

**Figure 8:**
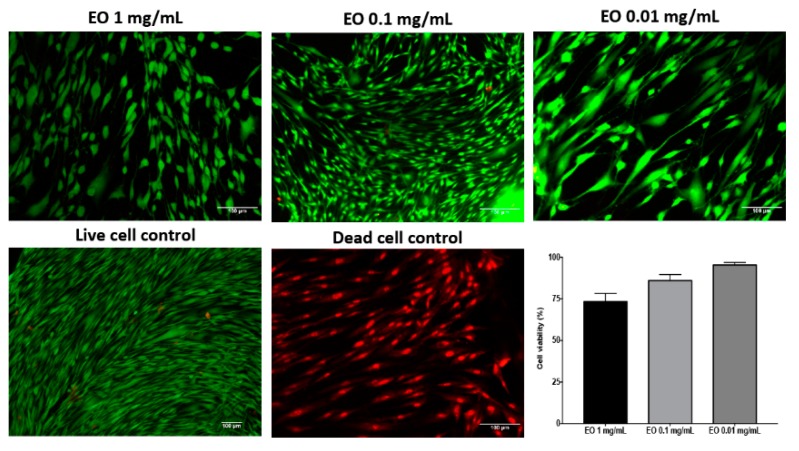
Cell viability test, life/death staining on fibroblast culture in monolayer, at 24 h post-stimulus with essential oil (EO) 1 mg/mL, 0.1 mg/mL, and 0.01 mg/mL.

Once the healing activity of EO of *B. morelensis* was tested, a series of in vitro tests were done to try to understand the mechanism of action. The cell viability test was performed in the life/death trial in monolayer, after 24 h of having applied the stimuli (EO 1 mg/mL, 0.1 mg/mL, and 0.01 mg/mL), finding that in the lower concentration, there was more cell viability since fibroblasts stimulated with EO 0.01 mg/mL showed a greater amount of green cells and fewer red nuclei (Figure 8). 

**Figure 9:**
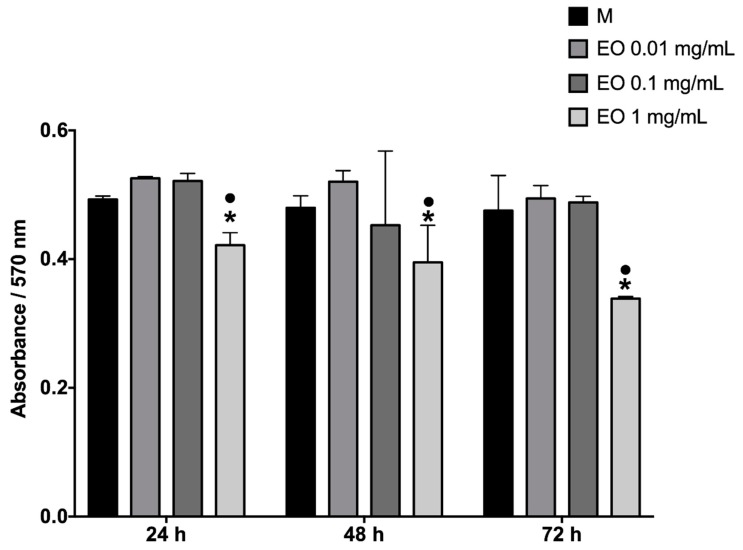
Cell proliferation assay of monolayer cultures. Resazurin absorbance indicated the proliferation of fibroblasts with absorbance control with medium plus Tween. The stimuli applied: M: DMEM media supplemented; EO 0.01 mg/mL; EO 0.1 mg/mL, and EO 1 mg/mL. *Significant differences with respect to M. ●Significant differences with respect to EO 0.01 (*p* < 0.05).

Cell proliferation was evaluated by the PrestoBlue assay, where the absorbance was directly proportional to cell proliferation. Cells were cultured for 72 h, stimulated every 24 h with EO (1 mg/mL, 0.1 mg/mL, and 0.01 mg/mL). It was evident that with a higher concentration of EO, there was less fibroblast proliferation; the bars corresponding to EO 1 mg/mL were the smallest (Figure 9).

**Figure 10:**
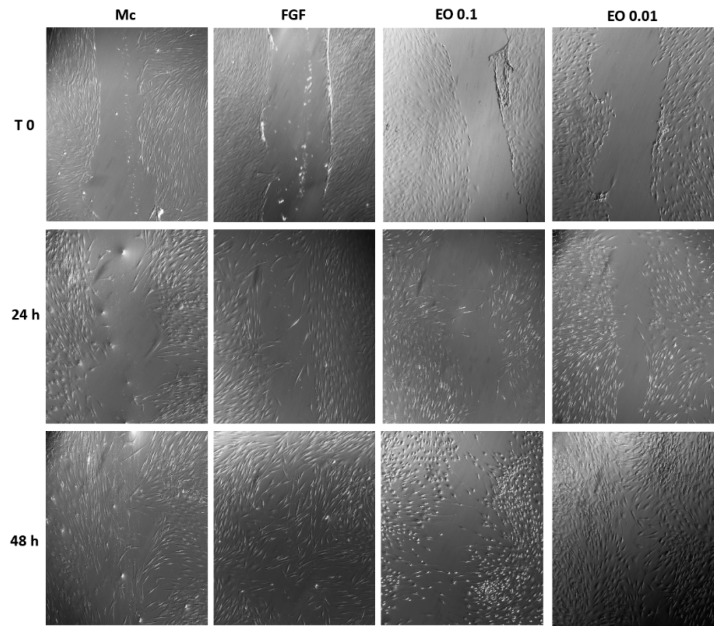
Cell migration test. A wound was simulated in the center of the cell monolayer and then the stimuli were applied: M: supplemented DMEM; FGF: fibroblast growth factor; EO: 0.1 and 0.01 mg/mL. The photographs corresponded to time 0 (immediately after inflicting the wound) at 24 h and 48 h after the application of stimuli.

Since proliferation experiments demonstrated that fibroblasts were cultured properly in the presence of EO, cell migration assays were carried out. Figure 10 shows the plates with confluent fibroblast culture, in which a line that simulated a wound in the monolayer was drawn. It was observed that at 24 h, the migration to the site of the wound was evident in the plaques where fibroblast growth factor (FGF) was applied, which stimulated the migration of these cells, while, in the plates treated with EO, the migration became noticeable until 24 h; likewise, in the plate with EO 0.01 mg/mL, more cells were observed, which also showed a better appearance in terms of shape and size (more similar to cell growth control).

## 3. Discussion

Studies of the *Bursera* species are very limited; the wound healing activity of the essential oil of *B. morelensis* was reported here for the first time.

The compounds that constitute the chemical mixture of the EO are responsible for their biological activities. The EO of *B. morelensis* used in this study is composed of 18 terpenes, of which probably *p*-menthane (SI 83%) is the main constituent. An isomer of *p*-menthane (*m*- menthane) has been reported in the EO of *Jatropha neopauciflora* (Pax), confirming its antibacterial activity against *Staphylococcus aureus* and *Vibrio cholera* [25]. The second compound in abundance is probably β-phellandrene (SI 68%), which has been identified in the EO of pine species, such as *Juniperus formosana* [19], as well as the EO of *Zanthoxylum bungeanum*, an oil that has potential in the treatment of psoriasis [26]. α-Pinene is the main constituent of the *Pistacia atlantica* resin, which has been proven to treat burn wounds, showing an increased concentration of basic fibroblast growth factor (bFGF) and platelet-derived growth factor (PDGF) and also increased angiogenesis [27]. In addition, α and β-pinene are present in the *Salvia officinalis* EO composition and have in vitro anti-inflammatory activity due to inhibited nitric oxide (NO) production in mouse macrophages [28]. In vitro testing found that caryophyllene and caryophyllene oxide inhibit the genotoxicity of a condensate of cigarette smoke [29]. Additionally, caryophyllene from the EO of *Aquilaria crassna* Pierre ex Lecomte has anticancer, antioxidant, and antimicrobial properties [30]. β-myrcene, orally administered to experimental animals, has demonstrated important protective activity in a model of the gastric ulcer [31]. Sabinene has shown strong anti-inflammatory activity mediated by the inhibition of NO production in macrophages [32], compared to α- and β-pinenes. Finally, p-cymene is one of the main compounds identified in thyme oil, and its ability to prevent lipidic peroxidation has been demonstrated [33]. These biological properties of the terpenes that constitute the essential oil of *B. morelensis* are the reason for the relevant wound healing activity that was observed in this essential oil. 

In the test to determine the cytotoxicity, doxorubicin was used as a positive control. Doxorubicin is one of the most effective anthracycline antibiotics, with a broad antitumor spectrum [34], and it has been recognized that various EO components act as multi-target molecules. With the aim of developing novel antitumor drugs, various EOs have shown high efficacy against human cancer cells and low toxicity to normal human cells. Some EO components, such as terpenes, have been found to be effective against a broad range of cancers, for example, geraniol, D-limonene, and other monoterpenes [35]. Our results indicated that the EO of *B. morelensis* could be used in topical application for wound because, at a concentration of 1.2 mg/mL, only 24% of peritoneal macrophages were inhibited, in comparison with doxorubicin, and the IC_50_ was = 0.85 µg/mL, confirming that EO at the concentration tested was not cytotoxic.

The healing process depends on the biosynthesis and deposition of collagen and its maturation [36]. Our results showed that both EO treatments had the better structure of scar tissue and a greater deposit of collagen. The antibacterial and antifungal activity of the EO of *B. morelensis* might be partly responsible for the results shown here because due to its lipophilic characteristics, the EO permeates the plasma membranes of both bacteria and fungi, generating ionic imbalances in membrane potential and even mitochondrial respiration, causing cellular collapse [9]. In our working group, it has been shown that the *B. morelensis’* EO alters the expression of the gene that codifies the integrin INT1p. This is very important since the integrins are known to be key in the adhesion of *Candida albican*; furthermore, EO inhibits the growth of the germ tube and causes the loss of the integrity of the cell membrane of this yeast [10].

On the other hand, we believe that regulation of the inflammatory response may occur by the wound repair since it has been shown that this EO has anti-inflammatory activity when used in topical form to treat plantar edema in rats [8]. Even more, several of its components have been identified as anti-inflammatory agents that inhibit the production of NO [28,32], while others increase the production of essential agents, such as FGF and PDGF, for wound repair and favor angiogenesis [28] and antioxidant activity, which may have a protective effect against the oxidative stress generated during the inflammatory stage [30,33]. Recently, it has been shown that α-phellandrene also inhibits leukocyte rolling and adhesion and production of the pro-inflammatory cytokines TNF-α and IL-6, as well as the degranulation of compound 48/80-induced mast cells. This suggests that α-phellandrene plays an important role as an anti-inflammatory agent through neutrophil migration modulation and mast cell stabilization [37]. 

Since the tests in the murine model demonstrated healing activity, a series of in vitro tests was performed, with an intention to approach the mechanism of action of the EO of *B. morelensis,* contributing to the healing process. The results of the cell viability test (life/death, Figure 8) coincided with the results of cytotoxicity in macrophages (Figure 2), in the sense of very low cytotoxicity of EO and its dependence on concentration. On the other hand, proliferation tests showed that EO did not stimulate fibroblast proliferation (Figure 9). Finally, fibroblast migration results suggested that EO promoted fibroblast migration (Figure 10).

Other natural compounds have been analyzed for their wound healing activity. For example, it is known that asiaticoside, a triterpene, is a major component in the extracts of *Centella asiatica* and is an element designated as a priority compound in the healing activity of this plant due to its antimicrobial activity and its ability to reduce lipoperoxidation levels and activate cells of the malphigian layer of the epidermis [37]. Moreover, it has been demonstrated that the essential oil of *Pinus pinaster*, whose main component is α-pinene, has antioxidant, anti-inflammatory, and wound repair activity, showing good tensile strength in the in vivo models; in the in vitro tests, this essential oil has shown a certain inhibitory capacity of the collagenase, elastase, and hyaluronidase enzymes, which are related to the remodeling of scars [38].

The set of results obtained in this work suggested that the probable mechanism of action of the EO of *B. morelensis*, during the healing process, is the promotion of the migration of fibroblasts to the site of the wound, making them active in the production of collagen and promoting the remodeling of this collagen since it is known that during the wound repair process, endothelial cells and fibroblasts migrate to the site and accumulate granulation tissues by depositing collagen and other extracellular matrices. During the final stages of repair, the fibroblasts reshape the collagen by producing matrix metalloproteinases (MMP) over several months [39].

## 4. Materials and Methods 

### 4.1. Plant Material

Young *B. morelensis* stems were collected from adult trees in the Cañada region of Teotitlan de Flores Magon, Oaxaca, Mexico, located 1234 m above sea level at latitude 18°08′39.6’’ and longitude 97°03′37.0’’, during March 2016. Samples were packed for further processing in the laboratory. Some of the material was deposited at the National Herbarium of Mexico (MEXU) at the Universidad Nacional Autonoma de Mexico and the herbarium IZTA at the Facultad de Estudios Superiores Iztacala (voucher specimens: IZTA 42123).

### 4.2. EO Extraction

The EO was obtained using the hydro-distillation method with 2000 g of the fresh plant, young stems. The distillation equipment consisted of a round-bottomed 1 L flask with a heating mantle (SEVPrendo, MC301-9, Mexico city, Mexico) attached to a double pass condenser, which was coupled to a cold-water circulator. For this, five extractions were made, each with 400 g of plant, and 500 mL of water was added. The EO was separated spontaneously from the aqueous phase by density differences; the resultant water phase was frozen at −18 °C in order to easily separate the EO residues by decantation in screw cap test tubes. The EO was stored in a glass vial in the dark at −18 °C until tested. The extraction yield of EO from young stems was calculated by the following equation:

% extraction yield (^g^/_g_) = a/b × 100 (1)

where a is the weight of EO obtained during the distillation process, and b is the weight of the plant material used for EO extraction.

### 4.3. Chemical Characterization

The analysis of the essential oil from *B. morelensis* was performed by gas chromatography-mass spectrometry (GC-MS, Agilent Technologies, Santa Clara, CA,) using a gas chromatograph (model 6850, Agilent Technologies, Santa Clara, CA, USA) coupled to a mass spectrometer (MS) (model 5975C, Agilent Technologies, USA), equipped with an RTX-50 column (30 m × 0.32 mm i.d. and 0.5 μm film thickness, Restek Corp, Bellefonte, PA, USA). Next, 1 μL of EO was injected by the split. The injector temperature was 280 °C. Peak area percentages were determined using RTE integrator software (Agilent Technologies, USA). The identification of the components was carried out by gas chromatography-mass spectrometry (GC-MS). Samples were ionized by electron impact at 70 eV, and the temperature achieved by the ionization source was 230 °C. An RTX-50 column (30 m × 0.32 mm i.d., 0.5 μm film thickness, Restek Corp, USA) was used. The separation conditions were an initial temperature of 70 °C for 2 min, then rising with 2 heating ramps, the first by 20 °C/min until reaching 250 °C, and the second by 8 °C/min until reaching 280 °C, which was maintained for 5 min. Helium was used as the carrier gas at a flow rate of 1 mL/min. The identification of chemical components was performed by the NIST Library Version 8.0 database (National Institute of Standards and Technology, Gaithersburg, MD, USA) [10].

### 4.4. Cytotoxicity

The cytotoxicity was determined using the crystal violet staining assay. It was performed with peritoneal macrophages from BALB/c mice seeded at 1.5 × 10^4^ cells/well treated with different concentrations of EO (from 1 mg/mL to 0.0004 mg/mL) in DMEM-F12/L glutamine (Biowest, Nuaillé, France) supplemented with 10% fetal bovine serum (Biowest, France) and 1% penicillin/streptomycin (Biowest, France), followed by incubation at 37 °C and CO_2_ 5% for 24 h. Subsequently, the medium was removed, and the remaining cells were stained at room temperature for 12 min with 50 μL/well crystal violet solution (1% crystal violet in 20% methanol/double-distilled water) and, consequently, washed several times with distilled water. The absorbance was measured at 595 nm. The relative viability was calculated as follows:

Relative viability = EA − background absorbance/UCA − background absorbance × 100(2)

where EA is experimental absorbance, and UCA is untreated control absorbance. The viability percentages were compared with those obtained using doxorubicin. The assays were performed in triplicate modified from [40].

### 4.5. Animals

Male CD-1 strain mice 6 to 8 weeks old were obtained from the animal laboratory facility of the FES-Iztacala, UNAM, Edo. Mex., Mexico. The animals, divided into experimental groups consisting of 6 mice each, were separately housed in ventilated cages under a controlled light cycle (12 h light/12 h dark) at standard room temperature (22–24 °C) and were allowed access to a conventional diet and tap water ad libitum. All guidelines for the care and use of animals were followed (NOM-062-ZOO-1999), approved by the Institutional Ethics Committee of the UNAM, Facultad de Estudios Superiores Iztacala (CE/FESI/052019/1295).

### 4.6. Skin Irritation Study

A preliminary skin irritation test was performed on the CD-1 male mice (*Mus musculus*). The back skin of 9 mice was depilated, and the mice were assigned to 3 groups (*n* = 3 mice in each group). Group 1, untreated healthy skin; Group 2, vehicle cosmetic grade mineral oil (Kamecare, Mexico); Group 3, 25% EO-treated skin. Twenty-four hours later, 10 μL of 25% EO and mineral oil was applied epicutaneusly every 12 h until 72 h, and the appearance of signs of irritation or erythema was recorded [37]. After this time, mice were sacrificed by CO_2_ chamber, and the dissected skin was processed for histological analysis.

### 4.7. Wound-Healing Efficacy

Mice were assigned to 5 groups (*n* = 6 mice in each group), and their back skin was shaved. Twenty-four hours later, mice were anesthetized by inhalation of isoflurane. Aseptic and antiseptic procedures were used for shaved skin, and a 1 cm incision was made. The groups were classified as follows: Group 1: untreated skin without wound or healthy skin (HS); Group 2: untreated wound (UW); Group 3: wound treated with Recoverón NC^®^ (Armstrong Lab, Mexico), as a positive control (C^+^); Group 4: 25% EO-treated wound (25EO); Group 5: 10% EO-treated wound (10EO). The wounds of Groups 4 and 5 were epicutaneously treated with 10 µL of the respective treatment, whereas the wounds of the control group were covered with Recoveron cream every 12 h. All treatments were applied over 10 days. After this time, the mice were sacrificed using a CO_2_ chamber. Immediately after the sacrifice, wound resistance to the tension was measured according to the tensiometric method [41]. The percentage of wound healing efficacy was calculated as:

% wound healing efficacy = GSS/GHS × 100(3)

where GSS is grams used to open scarred skin, and GHS is grams used to open healthy skin. 

### 4.8. Wound Contraction Model

To evaluate the wound contraction, the same groups of mice from the previous experiment were formed. In this procedure, a biopsy punch 5 mm in diameter, not deeper than the hypodermis, was performed, and the same treatment was applied every 12 h for 10 days. Every 2 days, the wound diameter was measured with a digital caliper (Mitutoyo, Tokyo, Japan), and the percentage of wound contraction was calculated considering the initial wound diameter as 100%.

### 4.9. Histopathological Observation

On day 10, the animals were sacrificed using a CO_2_ chamber. Skin specimens of wounds were obtained and immediately fixed in 10% buffered formaldehyde over 24 h at room temperature. Afterward, the skin samples were paraffin-embedded to obtain 4 μm-thick tissue sections, which were stained with hematoxylin and eosin (H&E) and Masson’s trichrome.

### 4.10. Isolation of Fibroblast

Fibroblasts were isolated from human skin, obtained by donation with written informed consent. The skin was taken from healthy voluntary donors, using a cylindrical scalpel for 5 mm biopsies, in septic and antiseptic conditions; the skin thus obtained was immediately deposited in Hank solution with antibiotic; later, in laminar flow hood, the skin samples were cut into smaller fragments, and each of these fragments was grown in Dulbecco Eagle Modified Low Glucose medium (DMEM-LG) supplemented with 10% fetal bovine serum (FBS) and antibiotics (100 U/mL penicillin, 100 mg/mL streptomycin, and 100 mg/mL gentamicin), all of Gibco BRL (Rockville, MD, USA), and incubated at 37 °C and 5% CO_2_. The culture medium was replaced every two days; after two weeks of culture, the explants (skin fragments) were removed. The fibroblasts were cultured to approximately 80% confluence, and the cells were separated with 0.05%/0.02% trypsin/EDTA and reseeded to generate sufficient cells for the following tests. 

### 4.11. Cell Viability and Proliferation 

Cell viability was analyzed in the monolayers of cultured fibroblasts through calcein and ethidium homodimer stain (LIVE/DEAD kit, Thermo Fisher Scientific, Waltham, MA, USA), according to the instructions of the manufacturer. For the assays, 5000 cells/cm^2^ were seeded in glass coverslips coated with poly-l-lysine (Sigma-Aldrich, St. Louis, MO, USA), cultured for 24 h with supplemented DMEM medium and stimulated with EO 1 mg/mL, EO 0.1 mg/mL, EO 0.01 mg/mL. Death control was obtained by treating cells with ethanol for 30 min before staining. Cells cultured with DMEM medium supplemented without EO were the control. Panoramic images (200 ×) were taken using a Nikon Eclipse 80i microscope (Nikon, Shinagawa, Tokyo, Japan) with the NIS-Elements F4 (Nikon) software (version Ver4.00.06, Tokyo, Japan). The total number of cells (live and dead) was counted with ImageJ software (version 1.52p, open source Java image processing program). The viability ratio was calculated according to the equation as follows:

(4) $$viability\;ratio=\frac{live\;cells}{live\;cells+dead\;cells}$$

For the cell proliferation assay, the scaffolds were incubated with PrestoBlue reagent ^®^ (Thermo Fisher Scientific) for 1 h, and then the supernatants were placed into 96-well plates. The absorbance of the content in each well was measured at a wavelength of 570 nm using a spectrophotometric plate reader (Thermo Multi skan Ascent Type 354). Each experiment was conducted three times [42].

### 4.12. Cell Migration

For this test, the fibroblasts were cultured in a six-well plate; once the monolayer was confluent, a wound was simulated in the center of the monolayer; for this purpose, a micropipette tip was used to draw a line that crossed the plate. Afterward, the following stimuli were applied: supplemented DMEM medium, 0.01 mg/mL EO, fibroblast growth factor (FGF) 10 ng/mL (positive control), and the negative control was medium without stimulation. In these crops, a wound was simulated. The response of the cells was monitored by observation under a microscope for 48 h [43].

### 4.13. Statistical Analysis

Results are expressed as the mean ± standard error of the mean. The analysis of the data was done using a one-way analysis of variance with a Tukey–Kramer multiple comparison posthoc test (*p* < 0.01) using GraphPad Prism 7 software (version 7.00, GraphPad Software, San Diego, CA, USA).

## 5. Conclusions

This work was the first report about the wound healing activity of the EO of *B. morelensis*. Our results indicated that this EO promoted the healing process by generating scars with effective tensile strength and accelerated wound closure by contributing to collagen deposition. The results also suggested that the essential oil of *B. morelensis* was involved in the healing process, stimulating the migration of fibroblasts to the wound site, with the consequent production of collagen. Additionally, due to its anti-inflammatory and antimicrobial capacity, it could be recommended for the treatment of minor wounds or where it is important to pay attention to the appearance and functionality of scars, such as eyelids and hands.

## Figures and Tables

**Table 1:** Chemical composition of *Bursera morelensis’* essential oil (EO).

Rt (min)	Compound	Abundance (%)	SI (%)
4.9	α-Phellandrene	0.80	90
5.029	α-Pinene	8.37	97
5.229	Camphene	0.13	96
5.421	Sabinene	3.54	93
5.51	β-Myrcene *	3.6	87
5.782	β-Phellandrene *	35.25	68
5.878	α-Terpinene	0.16	94
5.958	*p*-Cymene	2.1	97
6.063	*p*-Menthane *	38.41	83
6.255	γ-Terpinene	0.18	97
6.519	Terpinolene	0.3	97
7.409	Terpinen-4-ol	0.14	94
7.978	*p*-Menth-1(7)-en-2-one	0.34	93
9.317	β-Caryophyllene	5.19	99
9.557	α-Caryophyllene	0.28	99
9.71	Germacrene D	0.44	96
10.431	Caryophyllene oxide	0.26	98
10.912	β-Eudesmol	0.14	93

SI: similarity index or match between the library and mass spectra obtained. *: the identification of these compounds is partial because the SI is less than 90%.
